# Association of inflammatory bowel disease and related medication exposure with risk of Alzheimer's disease: An updated meta-analysis

**DOI:** 10.3389/fnagi.2022.1082575

**Published:** 2023-01-12

**Authors:** Yinghao Xing, Pei Li, Yuanyuan Jia, Kexin Zhang, Ming Liu, Jingjing Jiang

**Affiliations:** Department of Anesthesiology, Shengjing Hospital of China Medical University, Shenyang, China

**Keywords:** ulcerative colitis, Crohn's disease, Alzheimer's disease, dementia, meta-analysis, inflammatory bowel disease

## Abstract

**Background:**

Chronic systemic inflammation may be associated with neurocognitive decline, but the relationships between inflammatory bowel disease and related medications and the risk of Alzheimer's disease remain unclear.

**Methods:**

We performed a meta-analysis to evaluate the associations of ulcerative colitis, Crohn's disease and related medications with risk of Alzheimer's disease. We identified cohort and case-control studies by searching PubMed, Embase and Web of Science up to August 2022.

**Results:**

Seven eligible studies with 20,174 cases of Alzheimer's disease were included in the meta-analysis. Six studies reported the association between ulcerative colitis and risk of Alzheimer's disease; five studies reported the association between Crohn's disease and risk of Alzheimer's disease. Meta-analysis combining these studies did not reveal any significant association of ulcerative colitis or Crohn's disease with risk of Alzheimer's disease. The pooled relative risks were 1.16 (95%CI: 0.96, 1.41) and 1.17 (95%CI: 0.84, 1.62) for ulcerative colitis and Crohn's disease, respectively. High heterogeneity was detected across the studies. Of note, there was an inverse association between inflammatory bowel disease related medication exposure and risk of Alzheimer's disease. The pooled relative risk of three studies for Alzheimer's disease was 0.86 (95%CI: 0.75, 0.99). No publication bias was detected.

**Conclusion:**

This study does not support the association of ulcerative colitis and Crohn's disease with the risk of Alzheimer's disease. However, medications for the treatment of inflammatory bowel disease might be associated with a lower risk of Alzheimer's disease.

## Introduction

Alzheimer's disease (AD) is of increasing health concern with the global aging population (Alzheimer's Association., 2022). It is the most common cause of dementia, which accounts for an estimated 60–80% of cases (Alzheimer's Association., 2022). Other most common types of dementia include vascular dementia, Lewy body dementia, frontotemporal dementia, mixed dementia, and reversible causes (Robillard, 2007). The risk of AD is 60–80% dependent on heritable factors, such as carrying APOE ε4 allele (Livingston et al., 2020). Other risk factors are advanced age, an unhealthy lifestyle, and cardiovascular risk factors, etc (Hersi et al., 2017; Livingston et al., 2020; Litke et al., 2021). Inflammatory bowel disease (IBD), a chronic intestinal inflammatory condition, has become a global disease with increasing incidence in newly industrialized countries, and the prevalence is 0.3% in western countries (Ng et al., 2017; Mak et al., 2020). In recent years, the critical role of inflammatory processes in the pathogenesis of AD has been widely investigated. It is hypothesized that gut dysbiosis may increase inflammation and disrupt the blood brain barriers in IBD patients, leading to neuroinflammation *via* gut-brain axis and, consequently, increasing the risk of neurodegenerative disorders, such as Parkinson's disease and multiple sclerosis (Fu et al., 2020).

Emerging evidence from observational studies also indicated that IBD may be associated with risk of all-cause dementia (Sutton et al., 2019; Kim et al., 2022). Since the pathology of dementia is heterogeneous, understanding the roles of IBD and related medications with specific diseases causing dementia, particularly AD, is more significant to future basic research and precision prevention strategy. After summarizing results from a limited number of studies, recent meta-analyses found a higher risk of AD for IBD patients compared with general healthy population, suggesting a potential pathological detrimental effect of IBD on the development of AD (Liu et al., 2022; Szandruk-Bender et al., 2022; Zhang et al., 2022; Zuin et al., 2022). Of note, these reviews did not cover several large-scale population-based studies, which reported extensively inconsistent results (Jussila et al., 2014; Sutton et al., 2019; Aggarwal et al., 2022; Ronnow Sand et al., 2022). For example, two nationwide population-based studies with a total of 15,320 AD patients have been published after the recent reviews, and thus were not covered in these meta-analyses (Aggarwal et al., 2022; Ronnow Sand et al., 2022). Unexpectedly, a recent Mendelian randomization study indicated that genetically determined IBD was significantly associated with a decreased risk of AD, suggesting an unpredictably genetically protective effect of IBD on AD (Guo et al., 2021). However, other recent genome-wide association studies did not support a causal effect of genetically predicted AD on IBD (Adewuyi et al., 2022; Cui et al., 2022). In addition, IBD therapies, such as immunosuppressants and TNF-α blockers, may play an important role in the AD development by controlling the inflammation severity (Sutton et al., 2019; Aggarwal et al., 2022; Ronnow Sand et al., 2022). However, the association between medications for managing IBD and risk of AD was not evaluated in previous meta-analyses (Fu et al., 2020; Liu et al., 2022; Szandruk-Bender et al., 2022; Zhang et al., 2022; Zuin et al., 2022). Thus, we performed an updated systematic review and meta-analysis by summarizing the published data to evaluate the associations of IBD and related medication exposure with risk of AD.

## Materials and methods

### Literature search

The systematic review and meta-analysis followed the PRISMA guidelines (Liberati et al., 2009). PubMed, Embase and Web of Science databases were searched up to August 2022 without language restrictions, using the combination of the search term: (“Inflammatory bowel disease” OR “Ulcerative colitis” OR “Crohn's disease”) AND (“Alzheimer's disease” OR Dementia). We also searched the reference lists of relevant articles and google scholar for additional literature. Two researchers (KXZ and ML) independently performed the literature search and review. Disagreements were resolved by consensus in consultation with a third reviewer (JJJ). Details of the search strategy were shown in Supplementary Table S1.

### Inclusion and exclusion criteria

The inclusion criteria were as follows: (1) cohort or case-control studies, (2) Crohn's disease, ulcerative colitis or IBD-related medication treatment as exposure of interest, (3) risk of AD as outcome of interest, (4) studies reported risk ratio, hazard ratio, rate ratio, odds ratio or relative risk (RR) estimates to measure the associations of IBD and related medication exposure with risk of AD. When more than one study was conducted in the same study population, we selected the most recent study in the review.

### Data extraction

The following information were extracted: first author, year of publication, study location, study design, age at baseline, sample size, number of AD cases, exposure ascertainment, AD ascertainment, duration of follow-up, potential risk factors or confounders considered in the data analysis, the risk estimates with their 95% CIs. When multiple RRs were reported, we extracted the risk estimate that was adjusted for the largest number of possible confounders.

### Study quality assessment

We used the Newcastle-Ottawa Scale to evaluate the study quality. In this 9-star evaluation system, each cohort or case-control study was judged on the basis of three broad perspectives/domains: “Selection,” “Comparability,” and “Exposure/Outcome.” A study can be awarded a maximum of one star for each numbered item within the Selection and Exposure/Outcome categories. A maximum of two stars can be given for “Comparability” category to assess the control for confounders. Studies with more stars indicated higher quality.

### Statistical analysis

We used the RR as the risk estimate to measure the association. Fixed-effect model was used to pool the study-specific RRs when little heterogeneity was detected; otherwise, we performed meta-analysis by using a random-effect model (DerSimonian and Laird, 1986). The between-study heterogeneity was investigated with Cochran's *Q* test and the *I*^2^ statistic (Higgins and Thompson, 2002). The low, moderate, and high heterogeneity was defined as *I*^2^ values of 25, 50, and 75% (Higgins and Thompson, 2002). In sensitivity analysis, we excluded one study at a time and re-conducted the meta-analysis with the remaining studies to examine the influence of a single study on the overall result. Publication bias was assessed using Egger's test and funnel plot (Egger et al., 1997). The statistical analyses were conducted using Stata version 14.0 (StataCorp, Texas, USA).

## Results

### Study characteristics

**Figure 1:**
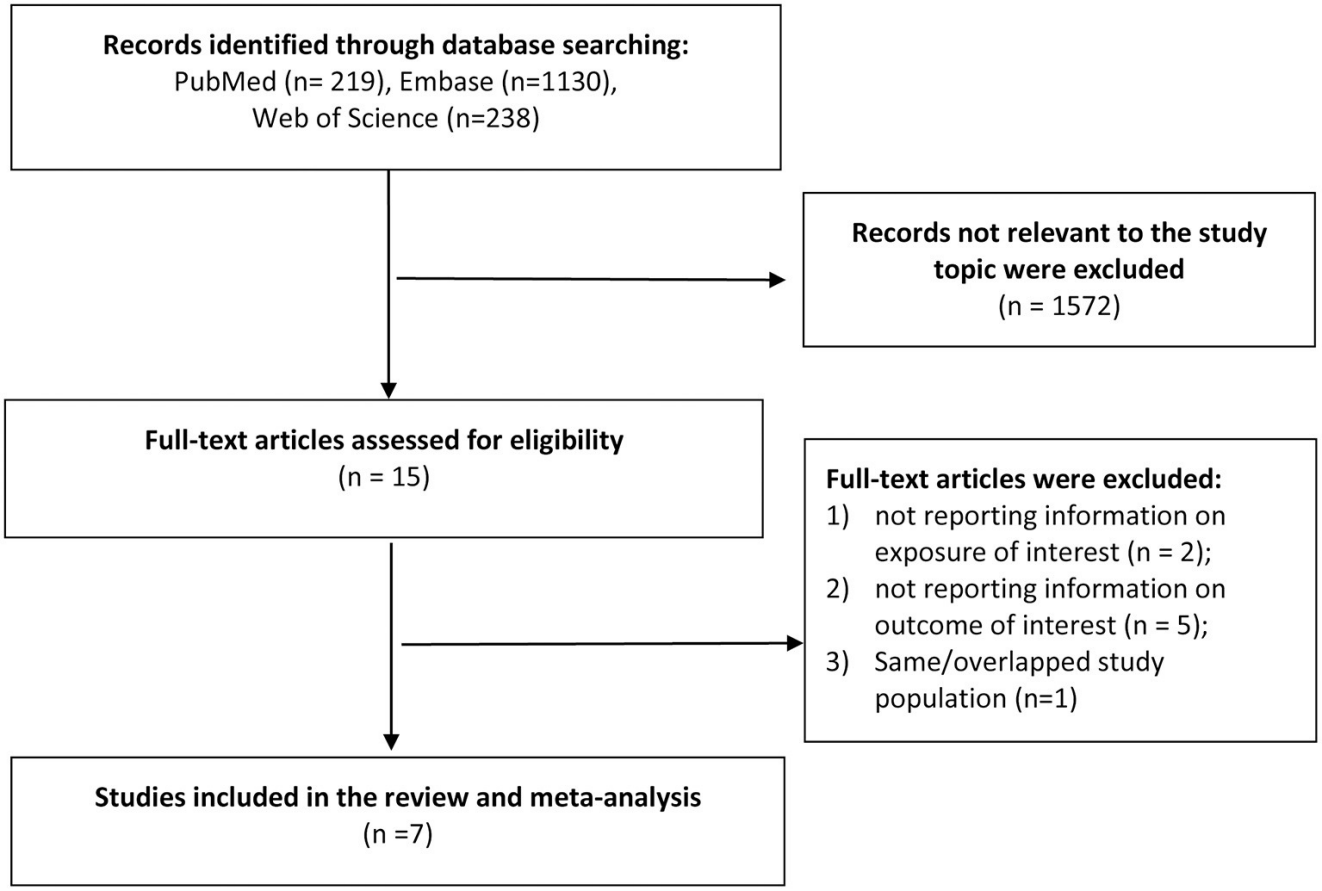
Flow diagram of study selection in the meta-analysis.

A total of 1,587 records was identified after the search of the three databases. After screening the titles and abstracts, we identified 15 most potential studies for full-text review. Finally, seven studies (six cohort studies and one case control study) were eligible in the meta-analysis (Jussila et al., 2014; Caini et al., 2016; Sutton et al., 2019; Zhang et al., 2021; Aggarwal et al., 2022; Kim et al., 2022; Ronnow Sand et al., 2022) (Figure 1). Three studies were conducted in the Europe, two in US and two in Asia. The study population covered all age groups. The exposure and outcome information were obtained from electronic health record, claims database, national registry or diagnosis by certified physicians. More details on the study characteristics were shown in Table 1. As assessed by Newcastle-Ottawa Scale, three cohort studies did not clearly report the exclusion of AD cases at baseline, which may prone to risk of bias. Most of the studies did not adequately adjust for possible confounders such as diet and exercise, and may expose to confounding bias (Supplementary Tables S2, S3).

**Table 1:** Characteristics of eligible studies in the meta-analysis.

**Study**	**Location**	**Study design**	**Age at baseline**	**Sample size**	**Number of events**	**Study period and follow-up**	**Source of exposure information**	**Source of outcome information**	**Confounder adjustments**
Ronnow Sand et al. (2022)	Denmark	Nationwide population-based cohort study	All age groups	61,895 UC and 27,090 CD patients; 884,108 matched controls without IBD	9,570 AD events	1977–2018; median follow-up time of 12.3 years	Danish national patient registry	Danish psychiatric central research registry	Age, sex, and region of residence, Charlson Comorbidity Index Score, chronic obstructive pulmonary disease, atrial fibrillation or flutter, obesity, diabetes (type 1 and 2), depression, hearing impairment and head trauma
Aggarwal et al. (2022)	US	National retrospective study with case-control design	Age ≥ 18 years	1,91,530 CD and 1,64,510 UC patients	5,750 AD events	1999–2020	Electronic health record	Electronic health record	Age, gender, race and traditional AD risk factors (hypertension, diabetes mellitus, hyperlipidemia, smoking, alcohol use and cannabis use)
Kim et al. (2022)	South Korea	Nationwide population-based cohort study	Aged ≥ 20 years with the mean age of 55.4 years	4,454 CD and 20,376 UC patients and 99,320 non-IBD controls	2,947 AD events	2009–2017; about 6 years follow-up	National health insurance service (ICD 10 code)	National health insurance service (ICD 10 code)	Age, sex, residential area and co-morbidities
Zhang et al. (2021)	Taiwan	Population-based cohort study	≥45 years with mean age of 61 years	1,158 UC and 584 CD patients and 17,420 controls	130 AD events	1998–2011; 16 years of follow-up	Diagnosis by board-certified gastroenterologists or colorectal surgeons	Diagnosis by board-certified psychiatrists or neurologists	Age, sex, enrolment time, dementia-related medical comorbidities, income level, and urbanization level of residence, a proxy for healthcare availability in Taiwan
Sutton et al. (2019)	US	National retrospective cohort study	Aged ≥ 55 years	24,057 IBD patients and 42,255 patients without IBD	1,689 AD events	2000–2019; >7 years follow-up	Inpatient, outpatient data and pharmacy claims	Inpatient, outpatient data and pharmacy claims	Age, race, index year, days in study, comorbidities (hypertension, hyperlipidemia, ischemic heart disease, type 2 diabetes, etc.), body mass index, medications (oral corticosteroids, methotrexate)
Caini et al. (2016)	Italy	Population-based cohort study	≥15 years old with median age of 40.5 years for UC patients and 33.9 for CD patients	689 UC and 231 CD patients	5 AD deaths	1978–2010; median follow-up of 22.8 years	Not reported	Regional mortality registry of Tuscany, the civil registry of the municipality of residence, death certificate	Age, gender and calendar time
Jussila et al. (2014)	Finland	Nationwide register-based cohort study	All age groups	16,649 UC and 5,315 CD patients	83 AD deaths	1987–2010; 10.8 years of mean follow-up	Identified by reimbursement code from National Health Insurance Scheme	National population register system; cause of death is based on the medical or forensic evidence	Age, gender and calendar time

AD, Alzheimer's disease; CD, Crohn's disease; IBD, inflammatory bowel disease; ICD, international classification of diseases, UC, ulcerative colitis.

### Ulcerative colitis and risk of AD

**Figure 2:**
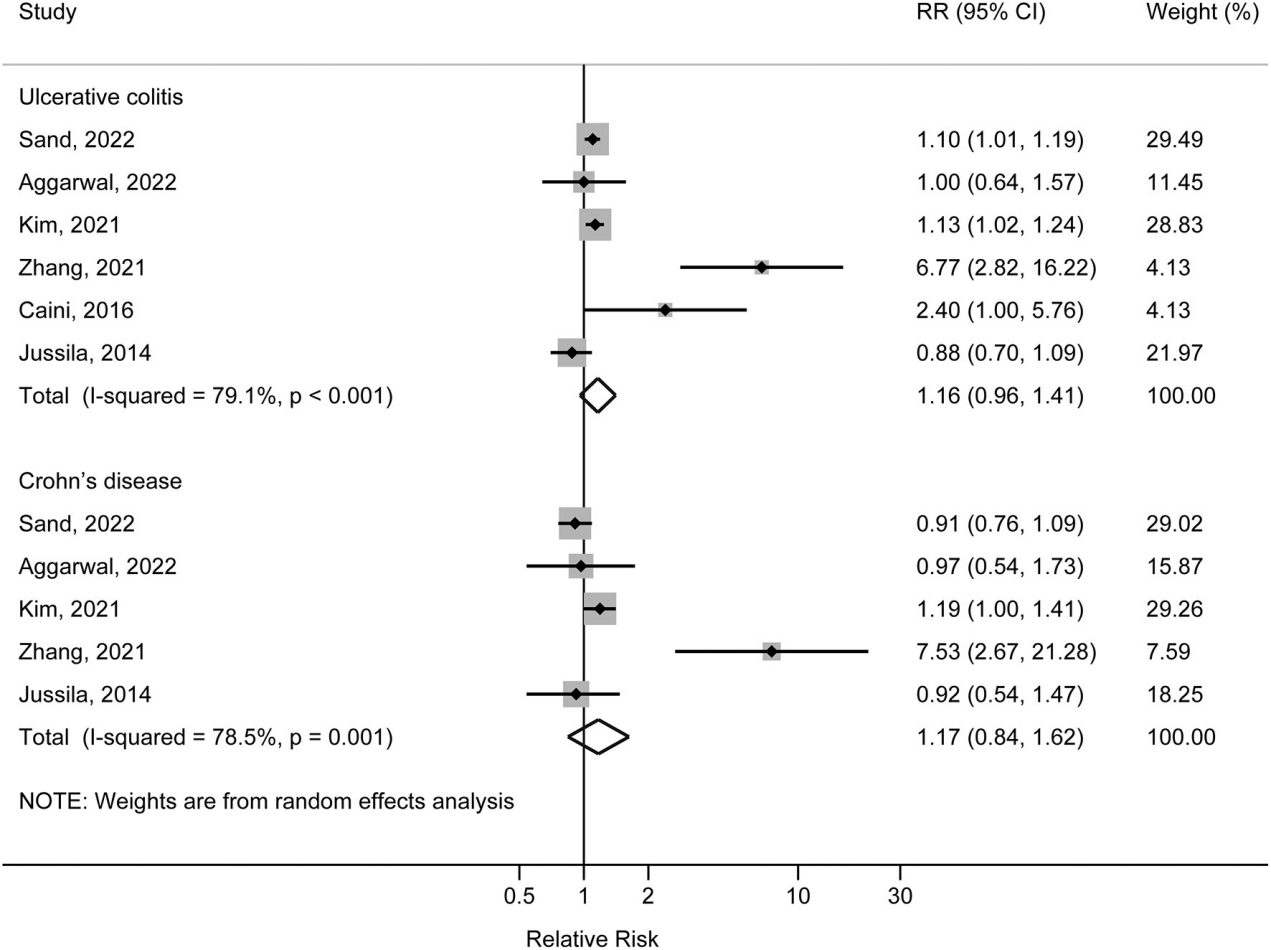
Forest plot of associations of ulcerative colitis and Crohn's disease with risk of Alzheimer's disease.

Six studies reported the association between ulcerative colitis and risk of AD. Four studies reported positive associations with point estimates of RR ranging from 1.10 (95%CI: 1.01, 1.19) to 6.67 (95%CI: 2.82, 16.22); the other two studies reported null association. Meta-analysis combining the data did not reveal an association between ulcerative colitis and risk of AD. The pooled RR was 1.16 (95%CI: 0.96, 1.41) with high heterogeneity across the studies (*I*^2^ = 79.1%, *P* for heterogeneity <0.001) (Figure 2). In sensitivity analysis, after the exclusion of Jussia 2014 study, the pooled result of the remaining studies was 1.27 (95%CI: 1.01, 1.56); the exclusion of other single study from the meta-analysis did not have substantial influence to the overall result. There was no indication of publication bias (Egger's test *P* = 0.335) (Supplementary Figure S1).

### Crohn's disease and risk of AD

Five studies reported the association between Crohn's disease and risk of AD. Zhang et al. (2021) and Kim et al. (2022) reported positive associations, with the corresponding RRs of 1.19 (95% CI: 1.00, 1.41) and 7.53 (95% CI: 2.67, 21.28); other three studies reported null associations with point estimates of RR ranging from 0.91 (95% CI: 0.76, 1.09) to 0.97 (95% CI: 0.54, 1.73). Meta-analysis combining the data did not reveal an association between Crohn's disease and risk of AD. The pooled RR was 1.17 (95% CI: 0.84, 1.62) with high heterogeneity across the studies (*I*^2^ = 78.5%, *P* for heterogeneity = 0.001) (Figure 2). In sensitivity analysis, exclusion of each single study from the meta-analysis did not have substantial influence to the overall result. There was no indication of publication bias (Egger's test *P* = 0.430) (Supplementary Figure S2).

### IBD-related medication exposure and risk of AD

**Figure 3:**
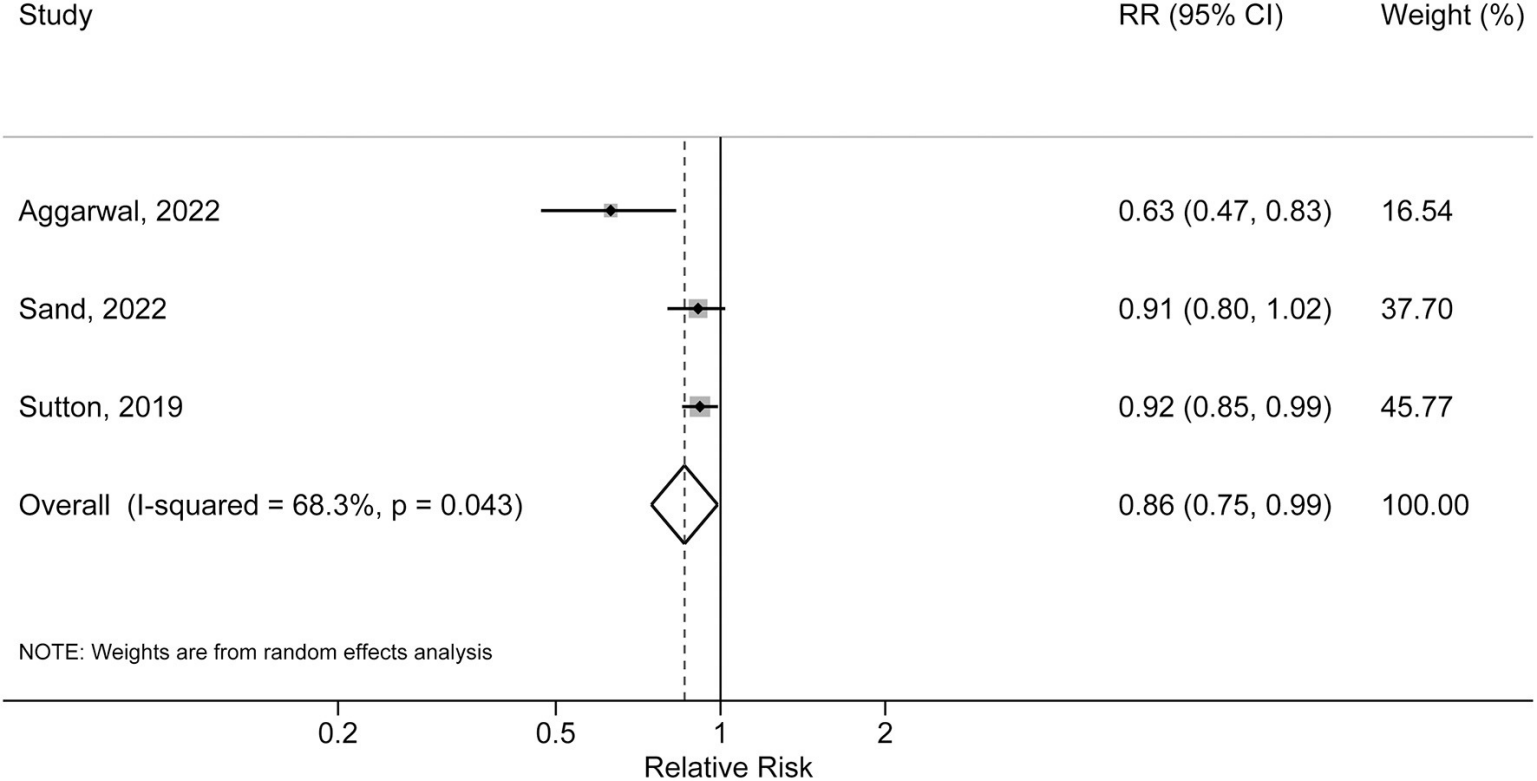
Forest plot of association between inflammatory bowel disease related medication exposure and risk of Alzheimer's disease.

Three studies evaluated the association of IBD-related medication exposure and risk of AD. Two studies reported a statistically significant inverse association between non-steroid medication exposure (immunomodulator, TNF-α and other biologics) and risk of AD, and one study did not detect significant association between steroid usage and risk of AD. Meta-analysis of these three studies indicated a pooled RR of 0.86 (95% CI: 0.75, 0.99; *P* = 0.043). Moderate heterogeneity was detected (*I*^2^ = 68.3%, *P* for heterogeneity = 0.043) (Figure 3).

## Discussion

After comprehensively reviewing all the published data from cohort and case-control studies, this updated meta-analysis did not support the association of ulcerative colitis and Crohn's disease with risk of AD. Of note, this is the first meta-analysis to evaluate the association between IBD-related medication and risk of AD. The result from the meta-analysis might indicate a possible lower risk of AD in IBD patients with a history of medication treatment compared with those without medication exposure.

Previous epidemiological studies observed a positive association between IBD and all-cause dementia (Bernstein et al., 2021; Zhang et al., 2021; Zingel et al., 2021; Ronnow Sand et al., 2022). A prior meta-analysis reported a 52% increased risk of all-cause dementia in IBD patients (Zuin et al., 2022). AD is the most common forms of dementia; however, unlike all-cause dementia, the association between ulcerative colitis and Crohn's disease and risk of AD varied remarkedly across the studies. A population-based cohort study in Taiwan demonstrated an unexpectedly 6-fold increase in risk of AD (Zhang et al., 2021). However, compared with general healthy population, AD may be more likely to be diagnosed in IBD patients due to surveillance bias. Furthermore, anti-inflammatory therapeutics and lifestyle confounders such as diet and exercise were not adjusted in the study, which may result in a biased estimate. In contrast, another national retrospective cohort study among U.S. Veterans observed a much lower rate of AD in IBD patients regardless of the thiopurine exposure (1.83 and 2.59 per 1,000 patient-years for thiopurine exposure and unexposed cohorts) compared with the non-IBD population (3.85 per 1,000 patient-years) (Sutton et al., 2019). However, this was a retrospective study of veteran patients comprising mostly of men. Therefore, the results may not be generalizable to non-veterans or to women. Furthermore, a recent Mendelian randomization study demonstrated that genetically determined IBD was significantly associated with a decreased risk of AD, suggesting an unpredictably genetically protective effect of IBD on AD (Guo et al., 2021). However, two recent genome-wide association studies did not support a causal effect of genetically predicted AD on IBD (Adewuyi et al., 2022; Cui et al., 2022). Several cohort studies also suggested null association; for example, a national cohort of US adults demonstrated that immune-mediated inflammatory diseases, as characterized by systemic inflammation, also did not have increased risk of AD (Booth et al., 2021). Likewise, the current updated meta-analysis did not support the association between IBD and risk of AD.

An important finding of the present study was that an IBD-related medication exposure was inversely associated with risk of AD. Similarly, a prospective cohort study evaluated the associations between several chronic inflammatory disorders including IBD and related drug therapies and risk of dementia (Dregan et al., 2015). It also suggested that anti-inflammatory therapies including glucocorticoid and non-steroidal anti-inflammatory drugs were associated with a lower risk of dementia across most disorders (Dregan et al., 2015). The evidence suggested that anti-inflammatory treatments might play an important role in reducing the AD risk *via* improving the IBD control and inflammation severity.

The study has several limitations. First, there was significant heterogeneity in this study. The heterogeneity may be due to the differences in study characteristics, such as study population and diagnosis criteria of AD, etc. Due to the limited number of included studies, we failed to perform the meta-regression to explore the sources of heterogeneity. Of note, we have conducted sensitivity analysis by excluding Zhang et al. (2021) study from the analysis. Although the overall results for ulcerative colitis and Crohn's disease did not change materially, the heterogeneity, as assessed by *I*^2^, decreased from 79.1 to 45.9% for ulcerative colitis and from 78.5 to 36.9% for Crohn's disease. Second, uncontrolled cofounding bias may distort the association. It was impossible to address the residual confounding effects inherent in the original studies without individual-level data. Third, the diagnosis of AD differed among studies and some degree of misclassification of AD may be also likely to occur. There might be high possibility of under diagnosis of AD, especially at the early disease stage. Likewise, the inaccurate diagnosis of ulcerative colitis and Crohn's disease may lead to some extent of exposure misclassification. Fourth, the inverse association between exposure to medications for IBD treatment and risk of AD should be interpreted with caution since only three studies were used in the meta-analysis. Further studies are still warranted to verify the findings. Lastly, although there was no indication of publication bias in this study, it may be inadequate to detect the publication bias when the sample size (number of studies) was small.

In conclusion, the current evidence does not support the association of ulcerative colitis and Crohn's disease with risk of AD. However, IBD-related medication exposure might be inversely associated with the risk of AD. Since high heterogeneity may exist across the studies, future well-designed, large-scale studies are still needed to explore the relationship between IBD and related anti-inflammatory medications and the risk of AD.

## Data availability statement

The data analyzed in this study is subject to the following licenses/restrictions: The datasets used and/or analyzed during the current study are available from the corresponding author on reasonable request. Requests to access these datasets should be directed to jjjiang@cmu.edu.cn.

## Author contributions

JJ developed the research design and had primary responsibility for the final content. PL, KZ, ML, and YJ collected and analyzed the data. JJ and YX interpreted the results and drafted manuscript. All authors critically reviewed and approved the manuscript.
