# Supplementary material for: Association of inflammatory bowel disease and related medication exposure with risk of Alzheimer's disease: An updated meta-analysis
Source: Front Aging Neurosci. 2023 Jan 12;14:1082575. doi: 10.3389/fnagi.2022.1082575 (PMC9878281; doi:10.3389/fnagi.2022.1082575)
Supplement: Supplementary file 1 [file Data_Sheet_1.docx]

**Table S1** Search strategy for the meta-analysis

| **Pubmed** | ("Inflammatory bowel disease"[All Fields] OR "Ulcerative colitis"[All Fields] OR "Crohn's disease"[All Fields]) AND ("Alzheimer's disease"[All Fields] OR ("dementia"[MeSH Terms] OR "dementia"[All Fields] OR "dementias"[All Fields] OR "dementia s"[All Fields])) |
| --- | --- |
| **Embase** | ('inflammatory bowel disease'/exp OR 'inflammatory bowel disease' OR 'ulcerative colitis'/exp OR 'ulcerative colitis' OR 'crohn disease'/exp OR 'crohn disease') AND ('alzheimer disease' OR 'dementia') |
| **Web of Science** | TOPIC: (“Inflammatory bowel disease” OR “Ulcerative colitis” OR “Crohn’s disease”) AND TOPIC: (“Alzheimer’s disease” OR Dementia)  Timespan: All years. Indexes: SCI-EXPANDED, SSCI, A&HCI, ESCI. |

**Table S2** Quality assessment for the cohort studies

| **Study** | **Representativeness of the exposed cohort** | **Selection of unexposed cohort** | **Ascertainment of exposure** | **Outcome not present at start of study** | **Comparability ^1^** | **Assessment of outcome** | **Follow-up long enough** | **Adequacy of follow up** |
| --- | --- | --- | --- | --- | --- | --- | --- | --- |
| Sand et al., 2022 | ⚝ | ⚝ | ⚝ | - | ⚝ | ⚝ | ⚝ | ⚝ |
| Kim et al., 2021 | ⚝ | ⚝ | ⚝ | ⚝ | ⚝ | ⚝ | ⚝ | ⚝ |
| Zhang et al., 2021 | ⚝ | ⚝ | ⚝ | ⚝ | ⚝ | ⚝ | ⚝ | ⚝ |
| Sutton et al., 2019 | ⚝ | ⚝ | ⚝ | - | ⚝ | ⚝ | ⚝ | ⚝ |
| Caini et al., 2016 | ⚝ | ⚝ | - | ⚝ | ⚝ | ⚝ | ⚝ | ⚝ |
| Jussila et al., 2014 | ⚝ | ⚝ | ⚝ | - | ⚝ | ⚝ | ⚝ | ⚝ |

^1^ A maximum of 2 stars could be awarded for this item. Studies that controlled for age and sex received one star, whereas studies that controlled for or considered most of the other important confounders such as lifestyle factors (smoking, physical activity, dietary factors etc.) and medication treatment etc. received an additional star.

^2^ A cohort study with a follow-up time more than 5 years was assigned one star.

**Table S3** Quality assessment for the case-control studies

| **Study** | **Adequate case definition** | **Representativeness of the cases** | **Selection of controls** | **Definition of controls** | **Comparability of cases and controls ^1^** | **Ascertainment of exposure** | **Same method of ascertainment for cases and controls** | **Non-Response rate** |
| --- | --- | --- | --- | --- | --- | --- | --- | --- |
| Aggarwal et al., 2022 | ⚝ | ⚝ | ⚝ | ⚝ | ⚝ | ⚝ | ⚝ | ⚝ |

^1^ A maximum of 2 stars could be awarded for this item. Studies that controlled for age and sex received one star, whereas studies that controlled for or considered most of the other important confounders such as lifestyle factors (smoking, physical activity, dietary factors etc.) and medication treatment etc. received an additional star.


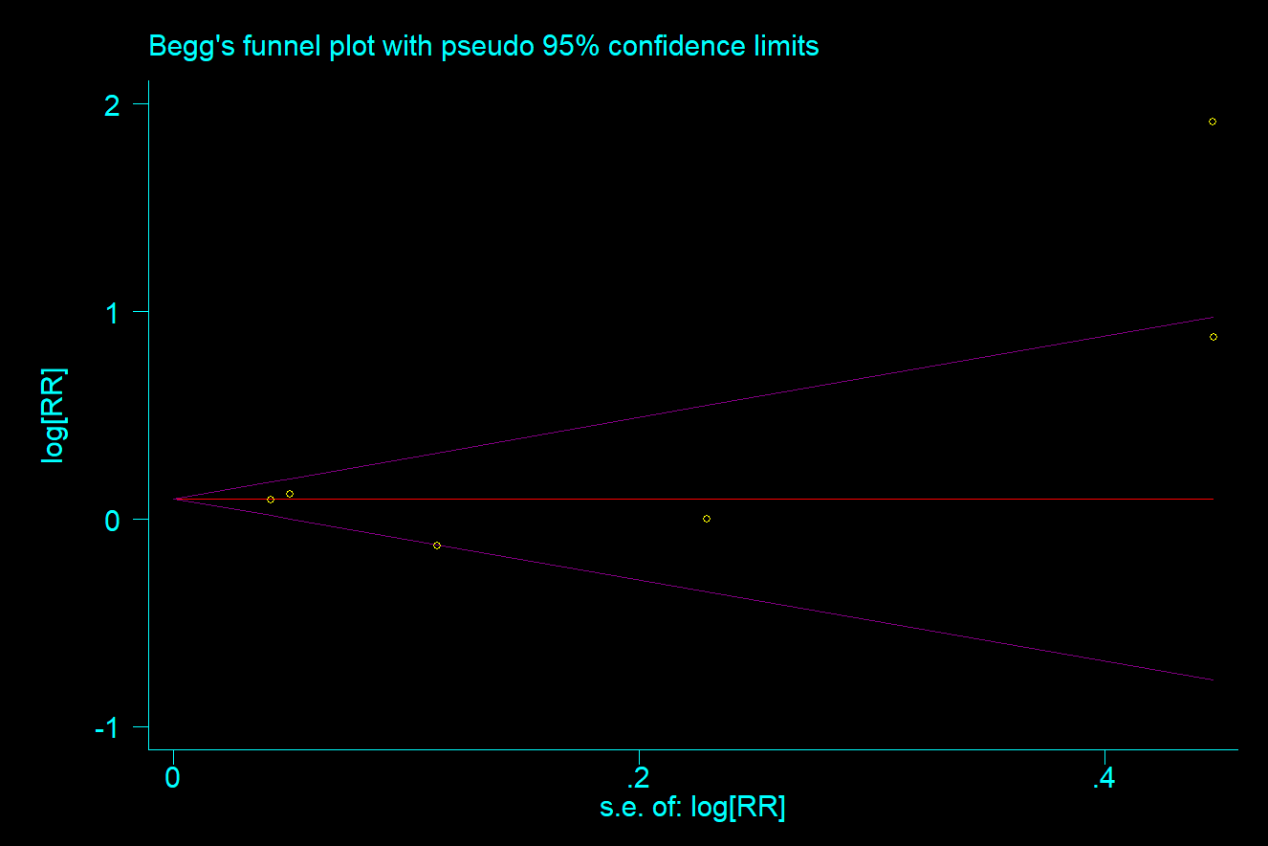


**Figure S1** Funnel plot of the association between ulcerative colitis and risk of Alzheimer’ s disease


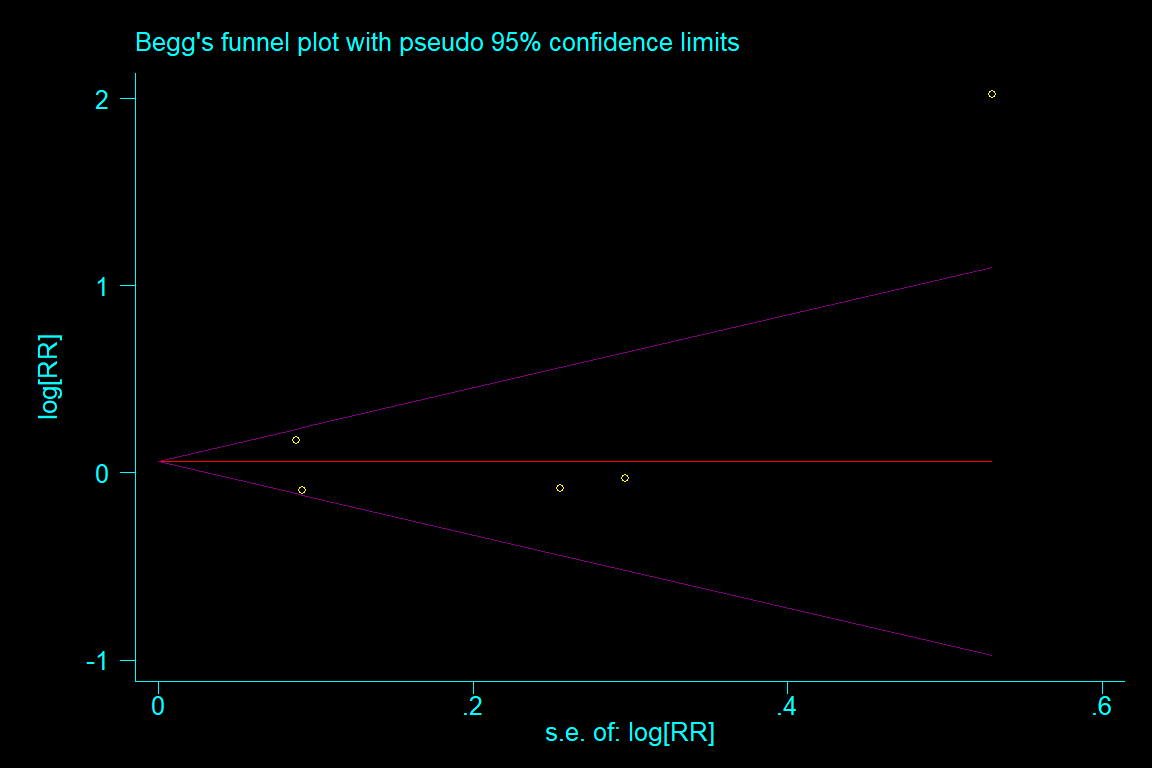


**Figure S2** Funnel plot of the association between Crohn’s disease and risk of Alzheimer’ s disease
